# Reducing Mental Health Stigma Through Identification With Video Game Avatars With Mental Illness

**DOI:** 10.3389/fpsyg.2020.02240

**Published:** 2020-09-09

**Authors:** Arienne Ferchaud, Jonmichael Seibert, Nicholas Sellers, Nivia Escobar Salazar

**Affiliations:** ^1^ School of Communication, Florida State University, Tallahassee, FL, United States; ^2^ College of Applied Studies, Florida State University Panama City, Panama City, FL, United States

**Keywords:** stigma, video games, transportation, identification, avatars

## Abstract

This study examines how playing a video game featuring a player-character with mental illness can positively impact mental illness stigma. We hypothesized that interactive gameplay would positively predict transportation into the story world. Then, transportation would predict identification with the main character. This identification should then reduce stigma in two ways: by lowering stereotyping and limiting participants’ desire for social distance. A two-factor, yoked experiment was designed utilizing *Hellblade: Senua’s Sacrifice*, a game praised for its accurate portrayal of psychosis. The main character, Senua, suffers from psychosis and must navigate her quest along with her own mental health. Players either played the first 45 min of the game or watched gameplay footage of other participants’ sessions. Transportation into the story world, identification with Senua, and the two aspects of stigma – stereotyping and social distance – were measured. Consistent with hypotheses, a structural equation model found an indirect path from gameplay to reduced social distance through first transportation and then identification. Players also reported higher levels of transportation than non-players, and that heightened transportation led to greater identification and then a lowered desire for social distance from the mentally ill. The indirect path to stereotyping was not significant. These results and implications are discussed in detail.

## Introduction

Stigma is a significant problem faced by those with mental illness. Being the target of mental health stigma can lead to negative impacts on the self, such as lowered self-esteem (Link et al., 2001) and life satisfaction. Stigma can also threaten a patients’ livelihood through discrimination in the workplace and in the realm of healthcare (Schulze and Angermeyer, 2003). Further, those who experience stigma – or who fear being stigmatized – are less likely to seek professional or medical help for the issues they face (Clement et al., 2015).

Due to negative and severe effects stigma can have on those with mental illness, researchers and advocates have attempted to create interventions to address the issue. However, many of these interventions can unintentionally have a boomerang effect, increasing the stigma they are attempting to reduce, either because of reactance or through the general resiliency of stereotypes (Corrigan and Penn, 1999).

Some research has suggested that embedding non-stigmatizing messages in entertainment media can increase the effectiveness of stigma reducing interventions due to the ability of narrative to bypass reactance and create links to characters that the viewer may not have interpersonal contact with (Moyer-Gusé, 2008). In this sense, video games may be uniquely positioned to deepen the connection with characters by placing the player directly into the body of a character with mental illness. Thus, rather than merely observing a character with mental illness, the player identifies with that character, feeling their thoughts, and experiencing life through the character’s lens.

The present study used an experimental design combined with structural equation modeling analysis to examine experiencing a mental illness through a character in a video game narrative, which can ultimately reduce mental health stigma through transportation and identification. We conducted a two-factor experiment, in which participants either played or watched gameplay footage of *Hellblade: Senua’s Sacrifice*, a game praised for its accurate and sensitive portrayal of individuals living with psychosis.

### Mental Illness Stigma

Numerous paradigms and perspectives have examined stigma. One such paradigm is the social cognitive approach, which suggests that stigma signals, or cues, lead to the construction of stereotypes, leading to discriminatory patterns of behavior (Corrigan, 2000). First, someone sees a person with mental illness exhibiting some signals, such as self-harm scarring or erratic behavior. This signal cues stereotypes, such as believing that those who have mental illness are dangerous. Based on this stereotype, the stigmatizing individual may engage in discriminatory behavior, such as distancing themselves socially from the person with mental illness.

According to Link et al. (2001), stigma occurs when different components co-occur. These components are labeling differences, associating differences with negative attributes, separating the “us” from “them,” and status loss/discrimination. Essentially, the stigmatized individual is recognized as being different and thus has stereotypes assigned to those differences. These stereotypes can result in the stigmatized individual being discriminated against in various ways, including a desire by the non-stigmatized group to distance themselves from the stigmatized individual, who makes up an out-group.

Stigma can have incredibly damaging effects on the stigmatized individual. Discriminatory behavior can result not only in social scorn but also loss of opportunities, including being released from employment (Rüsch et al., 2005). Further, stigma can be internalized, such that the individual holds stigmatizing beliefs about themselves. This can also be seen in that individuals with mental illness have been found to have lower rates of self-stigma in countries or regions that have less stigmatizing attitudes toward mental health (Evans-Lacko et al., 2013).

Further, since mental illness can be invisible, the stigmatized individual can shy away from behaviors that would mark them as a person with mental illness, which includes seeking professional help. Numerous studies have suggested that self-stigma and perceived stigma can both act as barriers to seeking help (Barney et al., 2006; Schomerus and Angermeyer, 2008; Eisenberg et al., 2009). Because of the negative impact of stigma, numerous researchers have explored how to reduce stigma through mass media. These interventions typically act as public service announcements or various methods of interpersonal contact with individuals suffering from mental illness and have had mixed success (Heijnders and Meij, 2006; Corrigan et al., 2012; Griffiths et al., 2014; Niederkrotenthaler et al., 2014).

In a study, Clement et al. (2013) showed that mass media interventions could potentially reduce prejudice against those with schizophrenia and major depression in a small to medium range. However, in terms of discrimination, there is still more research to do. Importantly, Clement et al. (2013) examined mass media interventions generally, including public service announcements, pamphlets, and brochures. They did not explore the role of commercial entertainment media in reducing stigma.

Research into the role of entertainment in reducing mental illness stigma has been mixed. Ritterfeld and Jin (2006) found that being entertained by a sensitive portrayal of mental illness led to higher educational value and thus stigma reduction. However, these positive effects only occur when the content is not stereotypical, else stigma can increase (Wahl and Lefkowits, 1989). For example, Rubenking and Bracken (2015) found that those who watched stigmatizing portrayals of mental illness reported greater stigma than did those in a control condition, who in turn reported greater stigma than those watching a sensitive portrayal. Thus, portrayals of mental illness need to be sensitive and nuanced if they are to reduce stigma and have positive societal effects.

Video games offer a unique avenue into the study of stigma reduction. While some research has suggested that contact – even non-face-to-face contact – with mentally ill individuals can be effective in reducing stigma, video games offer the opportunity to take contact further by handing the player direct control of the mentally ill character. While some gaming research has explored how playing with diverse others can decrease out-group bias (Adachi et al., 2014), less has been done to explore how experiencing gaming narratives with out-group members – in this case, someone with mental illness – can decrease bias, particularly.

### Transportation

When someone encounters a story, they may in some cases begin to feel as though they are themselves inside the world created by that narrative. Transportation theory (also described as narrative engagement or absorption) describes this process (Green and Brock, 2000). According to Green and Brock (2000), transportation occurs when a reader, viewer, or player disengages from the real world, and cognitive abilities are devoted to processing the story world. The world within the story becomes so salient that the outside world falls away while the individual focuses completely on the world created by the narrative.

Transportation is an effective mechanism for creating attitudinal change. The Extended Elaboration Likelihood Model (E-ELM) suggests that transportation into a narrative can result in positive responses to embedded persuasive messages, particularly by suppressing counterarguing against those messages (Slater and Rouner, 2002). Further, “based on identification and transportation’s ability to absorb audience members and encourage them to reevaluate their current perspectives and schemas, such experiences may be able to motivate viewers to positively reformulate their understanding of stigmatized others” (Chung and Slater, 2013). Essentially, those who become transported into a narrative surround themselves with events related to the target of stigmatization, thus forcing them to confront the nature of the stigma.

Of course, video games have an added advantage to other media in increasing transportation. Whereas, books force the reader to imagine the story world, and movies only show the narratives, video games allow players to explore on their own, deciding where they go and when. Additionally, players can embody their character in these spaces. Gee (2008) argues that video games can be used as a simulation of the human experience of embodied cognition. Players inhabit virtual characters, taking these characters on as surrogates and acting as though this character’s goals are their own. He further argues that digital characters have virtual minds and bodies and that when players inhabit these characters, they take these virtual minds and virtual bodies on as their own. Players use information from both the narrative and the game world itself to infer the state of their avatar’s mind and body. Players use this information to explain their characters’ actions within the virtual world.

This additional sense of control and the experience of embodying a character heighten transportation by easing the transition from the outside world to story world, while placing control over the story and its reality directly into the hands of the player (Green et al., 2004). Based on this line of reasoning, we propose:

Hypothesis 1: Playing a video game rather than watching will lead to an increase in transportation.

Of course, transportation is only possible when there is a narrative – and thus necessarily characters – to explore. Narratives in video games, while similar to traditional narratives, differ in that in most cases players take a direct role in controlling the actions of their character rather than being a passive observer. This could take the form of making choices which affect the narrative of the game, or in cases where narrative choice is not present, players still actively experience the story by leading the character along the journey, thereby exploring the world created by the video game by directly interacting with it.

### Identification

Identification, as discussed by Cohen (2001), refers to an imaginative process wherein an individual takes on the thoughts and perspectives of media characters. In essence, users adopt the media persona’s characteristics for the duration of the experience. While identifying with a character, individuals expand their own self-concept to include the media character, thus feeling as though they are one with the media character (Hefner et al., 2007).

This process of identification fundamentally differs between interactive and non-interactive media. In non-interactive media, such as books and film, the characters are distinct from the user, while the user may take the character’s perspective, they also realize that the character is a separate entity with their own thoughts, feelings, and behavior. This characterization of the identification is considered dyadic, as the user and the character form a dyad (Klimmt et al., 2009).

When the medium is interactive, however, the relationship between player and character is far closer in social distance. The player controls the character’s movements and actions, and sometimes even their words or feelings. Thus, the player truly feels as though they and the character they control are one entity, occupying a monadic relationship (Klimmt et al., 2009).

Of course, identification with the avatar presupposes that the player is transported into the story world in the first place. Becoming absorbed into a narrative allows players to bring themselves into the story and thereby experience the events of the plot alongside the characters. According to Slater and Rouner (2002), the degree to which a user takes on the perspective of the character highly depends on the degree to which the user can experience the story vicariously *through* the character. As the player and character travel through the plot together, the player is better able to take on the thoughts, feelings, and behaviors of the character in question. According to Green et al. (2004, p. 318), “central to the process of identification is the adoption of a character’s thoughts, goals, emotions, and behaviors, and such vicarious experience requires the reader or viewer to leave his or her physical, social, and psychological reality behind in favor of the world of the narrative and its inhabitants.” Thus:

Hypothesis 2: Transportation will positively predict identification.

Identification is also an important factor for stigma reduction through narrative means. In particular, being able to take on the perspective of a stigmatized character allows the user to humanize the stigmatized group. Chung and Slater (2013) found that perspective-taking specifically predicts the social acceptance of stigmatized groups. Igartua (2010) also found support for this link between identification and attitudinal change.

According to Moyer-Gusé (2008), identification can cause acceptance of story-related beliefs primarily because users lose part of their own self-concept while encompassing the other, lessening the likelihood that users will counter-argue the message. As users take on the perspective of the character, their own perspective – which may be skeptical of the message – is ignored. Further, identifying with characters who are in some way different from the user can serve to increase empathy about the real-world groups to whom those characters belong (Slater and Cohen, 2017). Additionally, identifying with a character from a stigmatized group helps to blur in-group, out-group distinctions, thereby reducing negative thoughts, feelings, and behaviors toward that group (Chung and Slater, 2013). Based on this line of theorizing and the idea that stigma consists of both stereotyping and the desire to separate from the stigmatized individual:

Hypothesis 3a: Identification will negatively predict stereotyping toward those with mental illness.
Hypothesis 3b: Identification will negatively predict the desire for social distance from those with mental illness.

## Materials and Methods

### Participants

Participants were recruited from a subject pool at a large university in the Southeastern United States. In exchange for their participation, individuals received course credit. The subject pool pulled from students in communication courses, including large introductory classes featuring students from a variety of majors.

A total of 207 participants completed the questionnaire. However, seven participants turned in missing data for the mediating variables of interest (e.g., failing to answer questions about identification or transportation) and were excluded. Additionally, one participant missed an entire section of the questionnaire and was excluded. A missing value analysis indicated eight values missing. These values were found to be missing completely at random (MCAR) according to Little’s MCAR test, *χ* ^2^ = 285.05, *df* = 253, *p* = 0.075. Because of the low number of missing cases and the fact that they were considered to be MCAR, expectation-maximization was utilized to impute missing data. This resulted in a total *N* of 198. Participants ranged in age from 18 to 35, with a mean age of 20.42. Participants were mostly White (71.7%), followed by Hispanic/Latino (20.7%) and Black (13.1%), with participants able to choose more than one ethnicity. The majority (66.7%) were female, with 32.8% identifying as male and one participant identifying as neither male nor female.

### Design and Stimulus

The study design was a two-condition, randomized design comparing treatment and control and was approved by Florida State University’s Institutional Review Board prior to its being conducted. After recruitment undergoing the informed consent process, participants were assigned to one of two conditions: a treatment condition where they played a video game featuring a protagonist with mental illness, and a control condition where they merely watched someone else play the same game. The first thing that participants were shown when starting the game was the following disclaimer:

Warning: This game contains representations of psychosis. People with experience of psychosis as well as professionals in psychiatry have assisted in these depictions. Some may find these depictions disturbing, including those who, themselves, may have had similar experiences. If you would like to find out more about psychosis and mental health difficulties visit: www.hellbladehelp.info. This game also includes violent scenes that some may find distressing (Ninja Theory, 2017).

The conditions were fully yoked; that is, participants who watched gameplay footage were watching recordings of other participants in the study. Therefore, there should not be differences in the amount of story consumed between the two conditions. Upon completion of either a playing or viewing session, participants took a survey on their assigned computer that included the measures described below.

The video game chosen for the study is *Hellblade: Senua’s Sacrifice*. Set in an age of Vikings, *Hellblade* follows the title character, Senua, on a vision quest (Ninja Theory, 2017). Developed along with neuroscientists and those who experience psychosis, *Hellblade* places depictions of mental health issues centrally in its story as Senua battles for the soul of her departed lover (Ninja Theory, 2017). Senua herself suffers from psychosis and must contend with her own mental illness along with the challenges presented by her quest. *Hellblade* has been recognized for these depictions, winning a *BAFTA* award for “Game Beyond Entertainment” and a *The Games Award* prize for “Games for Impact” (Ninja Theory, 2017).

*Hellblade* was designed in conjunction with noted neuroscientist and mental health experts and people suffering from psychosis, with the express intention of creating an accurate and respectful depiction of the types of hallucinations common to those suffering from psychosis (Fordham and Ball, 2019).

A case study of *Hellblade*, done by (Fordham and Ball (2019, p. 8) found the game incorporated psychosis throughout both the narrative and gameplay aspects of the game. For instance, the game utilized binaural 3D microphones to give the illusion that the protagonist is hearing voices from different directions, leading experts in auditory hallucinations to argue that the game “is one of the best representatives of these experiences.”

Thus, through its thoughtful development and critical acclaim, *Hellblade* is uniquely positioned to help depict mental health issues.

### Independent Variable

The independent variable, in this case, is the manipulation of either playing the above-described video game or simply viewing gameplay footage of the game. If assigned to the playing condition, participants were asked to play the game on a PC in the lab for approximately 45 min, with their gaming performance recorded. Those assigned to the viewing condition watched the recorded footage of other participants playing. Recordings were done locally on computers in the lab, viewed and then removed from rotation when new gameplay recordings became available. In this way, the participants, both creating and viewing the recordings, as well as the recordings themselves, were randomly assigned.

### Measures

All items were measured using seven-point Likert-type scales ranging from 1 (Strongly Disagree) to 7 (Strongly Agree) unless otherwise noted. While measures were adapted and not used verbatim, we only altered items enough to make them fit the context of the game. For example, when asking about identification, we inserted Senua’s name so that participants could visualize the character as they answered the questions. Table 1 shows means and standard deviations for all measured variables.

**Table 1:** Means and standard deviations for all variables.

Variable	*M* (1–7)	*SD*
Identification	2.59	1.18
Transportation	4.09	1.30
Stereotyping	3.00	0.93
Desire for social distance	2.74	1.18
Contact with mentally ill	4.02	1.21
Perceived difficulty	3.92	1.97

#### Dependent Variables

Stigma against those with mental illness was measured in two ways. First, nine items adapted from Cohen et al. (2018) assessed the degree to which individuals would stereotype those with mental illness (adapted from Corrigan et al., 2006). Items included “Those with mental illness are to blame for their own problems” and “Those with mental illness will NOT recover or get better.” These items were used instead of other validated scales because they best fit the context of the present study. The resultant index was found to be reliable with a Cronbach’s alpha of 0.82.

Additionally, the desire for social distance from those with mental illness made up the second dimension of stigma. Six items were taken from Martin et al. (2000). The items used on the scales asked participants’ willingness to engage in certain behaviors. Items included “How willing would you be to spend an evening socializing with a person described as having a mental health problem?” and “How willing would you be to make friends with a person described as having a mental health problem?” The created index was reliable (*α* = 0.89).

#### Mediating Variables

Transportation was measured utilizing narrative engagement scale of Busselle and Bilandzic (2009). The transportation scale created by Green and Brock (2000) was not utilized in the study because it tends to work best for written narratives, due to the specific items on the scale (Bezdek and Gerrig, 2017). The narrative engagement scale consists of 12 items, including “During the game, my body was in the room, but my mind was inside the world created by the story” and “The story affected me emotionally” (*α* = 0.87).

Identification was measured with 16 items adapted from Player Identification Scale of Van Looy et al. (2012). Sample items include “If I could become like Senua, I would” and “Senua is an extension of myself.” The scale was found to be reliable with a Cronbach’s alpha of 0.93.

#### Covariates

Given that difficulty may have an effect on the dependent or mediating variables, it is important to measure participants’ perceived difficulty of the game and control for its effect in the model. The perceived difficulty was measured using a single item created for the study: “This game was difficult.”

Research has suggested that personal contact can reduce stigma (Alexander and Link, 2003; Couture and Penn, 2003). Therefore, we also measured contact utilizing eight items from Trute et al. (1989). These items included “I have received some formal education regarding mental health” and “I currently have or in the past have had professional help for mental problems” and were reliable with a Cronbach’s alpha of 0.71.

## Results

To test the proposed model, a structural equation model was constructed in two stages. First, a measurement model was calculated utilizing all variables of interest (e.g., experimental condition, transportation, identification, social distance, stereotyping, along with difficulty and contact). All items within subscales in the model (e.g., items predicting wishful identification, similarity identification, and embodied presence in the avatar identification scale) were correlated with one another. Additionally, all items with very low factor loadings (e.g., <0.40) were dropped, resulting in the removal of the entire narrative understanding subscale of the narrative engagement scale and one item from the stereotyping scale. While the resultant model had significant Chi-square, *χ* ^2^ = 1696.005, *df* = 969, *p* < 0.001, other indicators of model fit pointed toward an adequate fit, RMSEA = 0.06, 90% CI (0.06, 0.07), standardized RMR = 0.09. See Table 2 for factor loadings.

**Table 2:** Final CFA factor loadings.

Label		Loadings
Contact with mental illness (*α* = 0.71)		
C1	I have lived or worked close to a mental health facility.	0.43
C3	I am currently working with or in the past have worked with a coworker having mental health problems.	0.53
C5	I currently have or in the past have had professional help for mental problems.	0.49
C6	A member of my family currently has or in the past has had mental problems.	0.56
C7	I have received some formal education regarding mental health.	0.45
C8	I have read factual information or seen factual TV programs concerning mental health.	0.43
Transportation (*α* = 0.86)		
T4(R)	I found my mind wandering while the game was on.	0.52
T5(R)	While playing, I found myself thinking about other things.	0.53
T6(R)	I had a hard time keeping my mind on the game.	0.59
T7	During the game, my body was in the room, but my mind was inside the world created by the story.	0.75
T8	The game created a new world, and then that world suddenly disappeared when the game ended.	0.52
T9	At times during the game, the story world was closer to me than the real world.	0.61
T10	The story affected me emotionally.	0.61
T11	During the game, when a main character succeeded, I felt happy, and when they suffered in some way, I felt sad.	0.70
T12	I felt sorry for some of the characters in the game.	0.43
Identification (*α* = 0.93)		
I1	Senua is similar to me.	0.71
I2	Senua resembles me.	0.76
I3	I identify with Senua.	0.81
I4	Senua is like me in many ways.	0.86
I5	Senua is an extension of myself.	0.83
I6	I would like to be more like Senua.	0.65
I7	If I could become like Senua, I would.	0.66
I8	Senua is an example to me.	0.81
I9	Senua is a better me.	0.70
I10	Senua has characteristics that I would like to have.	0.58
I11	When I am playing, it feels as if I am Senua.	0.54
I12	I feel like I am inside Senua when playing.	0.54
I13	In the game, it is as if I become one with Senua.	0.55
I14	When I am playing, I am transported into Senua.	0.51
I15	When playing, it feels as if Senua's body becomes my own.	0.56
I16	In the game, it is as if I act directly through Senua.	0.50
Stereotyping (*α* = 0.82)		
S1	Those with mental illness are to blame for their own problems.	0.44
S2	Those with mental illness are dangerous.	0.84
S3	Those with mental illness are morally weak.	0.66
S4	Those with mental illness are unpredictable.	0.62
S5	Those with mental illness are reckless.	0.77
S6	Those with mental illness engage in high risk behaviors.	0.62
S8(R)	Those with mental illness make important contributions to society.	0.46
S9(R)	Those with mental illness live a good and fulfilling life.	0.46
Desire for social distance (*α* = 0.89)		
SD1(R)	How willing would you be to…move next door to a person described as having a mental health problem?	0.81
SD2(R)	…make friends with a person described as having a mental health problem?	0.86
SD3(R)	…spend an evening socializing with a person described as having a mental health problem?	0.84
SD4(R)	…have a person described as having a mental health problem start working closely with you?	0.86
SD5(R)	…have a group home for people described as having a mental health problem?	0.46
SD6(R)	…have a person described as having a mental health problem marry into your family?	0.81

(R) Indicates a reverse-coded item. Cronbach’s alphas indicate reliability after items low-loading items were dropped. Chi-square, *χ* ^2^ = 1696.005, *df* = 969, *p* < 0.001, RMSEA = 0.06, 90% CI (0.06, 0.07), and standardized RMR = 0.09.

**Figure 1:**
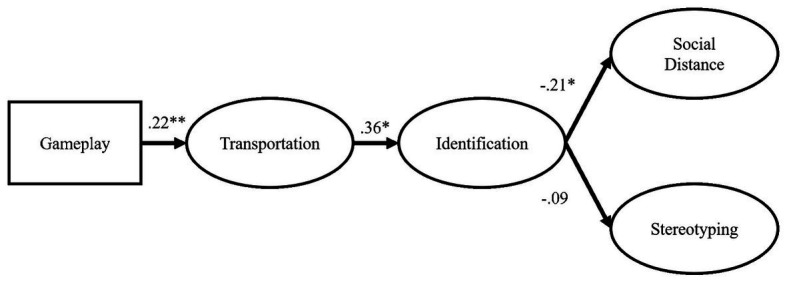
SEM path model. Items and covariates (i.e., difficulty and contact) are not pictured here. Paths represent standardized regression weights. ^*^ *p* < 0.05, and ^**^ *p* < 0.01.

Then, the structural model was constructed. This model exhibited adequate fit as well, Chi-square, *χ* ^2^ = 1843.960, *df* = 1,028, *p* < 0.001, RMSEA = 0.06, 90% CI (0.06, 0.07), standardized RMR = 0.11. As shown in Figure 1, playing the game (as opposed to watching) increased transportation, which increased identification. Identification then *decreased* the desire for social distance. The same pattern did not hold for stereotyping, however.

One potential concern here was that, although players encountered relatively few enemies in the first portion of the game, violent combat was a part of the experience. This violence is a typical stereotype for those with mental illness, which may have implications for how the stereotyping variable behaved. To check for this, we removed one item from the model about those with mental illness being dangerous; this change did not alter the results. As a result, we have reported the original model with the violence item variable included.

## Discussion

The goal of this study was to examine how playing video games featuring characters suffering from mental illness may ultimately reduce stigma through transportation and identification.

Consistent with the hypothesized model, playing a video game was associated with an increase in transportation. Those who played the game reported greater feelings of being lost inside the story world than did those who merely watched gameplay. This feeling of being totally involved in the world of the story appears to have increased the connection players felt with the main character. As they were able to move around and explore the world in Senua’s shoes, they were able to get a better feel for her thoughts, feelings, and behaviors. This heightened identification seems to have led to a reduction in desire for social distance from mentally ill others. These results are especially meaningful, given that Senua’s mental illness is psychosis, an illness that is hugely stigmatized, more so than other illnesses such as depression or anxiety. It seems that, by experiencing life as someone afflicted with this disorder, players were able to incorporate the mental illness to their own self-concept through the process of identification, therefore, reporting less desire to keep those with mental illness away. Notably, the measurement for social distance did not specify a diagnosis, indicating that playing as a character with this illness helped with perceptions of mental illness overall.

These results did not hold for stereotyping, which is another critical aspect of stigma. Identifying with Senua was not associated with changes in held stereotypes about mental illness. This is a somewhat counterintuitive finding since stereotyping is often found to be easier to shift than the desire for social distance (see Hoffner and Cohen, 2012; Wong et al., 2017). A possible explanation is that, when individuals incorporate a character’s attributes – in this case, mental illness – as part of their own self-concept through identification, this expansion of the self to include the other in the in-group does not necessarily limit the possibility of stereotyping that new perception of the in-group. In other words, even though participants can take on Senua’s thoughts, attitudes, and behaviors as though they were the players’, they may still stereotype their own self-concept. Interestingly, removing the stereotyping items related to violence did not change the overall model as reported. These results indicate that something other than violence is driving the stereotyping result. The specific mechanisms by which stereotyping may be lowered must be further explored in future study.

Additionally, transportation into the narrative appears to be a strong predictor of identification. This finding is consistent with previous literature, such as the model proposed by Brown (2015). Becoming personally involved in the narrative allows participants to become involved with the lives of the characters at play. Given that, as Green et al. (2004) argue, identification requires the individual engaging with the narrative to adopt the character’s motivation, actions, behaviors, and experiences, the findings of this study support this conceptualization.

It is important to note that identification was not, on average, particularly high (*M* = 2.69, *SD* = 1.18). As Chung and Slater (2013) found, it seems possible that fostering a sense of identification with a member of a highly stigmatized group is difficult. That said, even this low level of identification was significantly associated with lower levels of desire for social distance. Moreover, the results could also indicate that the identification may have different outcomes when the participants are watching the game instead of playing. The participants who watched might not have the same experiential value of identification compared to those who played. This condition may mirror the experience of watching gameplay videos online; the watcher is not directly interacting with the character but is forced into a further layer of distancing brought on by the presence of a third party – the player. According to Zillmann, “at least in non-interactive entertainment such as watching television – media users keep a distance between themselves and a character on the screen,” as cited in (Hefner et al., 2007, p. 40), which was supported here.

Participants who watched Hellblade: Senua’s Sacrifice may not have had the opportunity to identify themselves with the character, at least not to the same degree as those who played the game; this could be due to the game itself. “Hellblade: Senua’s Sacrifice” is a game that is not categorized as, e.g., an action game, simulation game, war game, or e-sports game, etc. Those who watched maybe have not been motivated since the purpose of the game is different from the best selling and the most played video game. “When identifying with a character or role offered by the game, players change their self-concept by adopting relevant attributes of the character, for instance, they perceive themselves as more courageous, heroic, and powerful during identification with a soldier” (Hefner et al., 2007, p. 39).

### Theoretical Implications

The results of this study hold critical theoretical implications, particularly in the realm of mental illness stigma. Whereas, some preliminary work has begun to explore the role of entertainment media in stigma reduction, the current study extends this work by explicitly focusing on video game contexts. In essence, playing narrative-heavy video games seems to be a potential method to reduce social distancing, mainly through engagement and identification.

There are also implications for the study of entertainment media, particularly in narrative processing. By controlling a non-stereotypical character in a rich narrative space, participants reported an increased sense of transportation into the narrative constructed by *Hellblade*, and thus possibly feeling a stronger connection to Senua. These findings highlight the unique ability of video games to foster this sense of transportation – and thus, identification – through the interactivity afforded by the medium. This work offers an important step in further exploration of video games for social good.

### Practical Implications

Given that participants played a commercially available game and still showed a slight reduction in social distancing, it seems that video games can reduce stigma as long as the portrayal shown in the game is not stigmatizing. Great care was taken by the development team to ensure their portrayal of psychosis was accurate and sensitive. As a result, players who controlled Senua on her quest showed a reduction in the social distance aspect of stigma.

This finding is important for two reasons: first, it illustrates that commercially available games can affect change in comparison to simply watching, even when that may not be the developers’ primary purpose. Second, the results of this study may help create games designed explicitly for stigma reduction in the future.

Furthermore, it is also important to mention that some players are interested in watching others playing video games. According to Kaytoue et al. (2012, p. 1181), “casual players were found to prefer watching professional gamers rather than playing the game themselves.” If the players show skills during the game and prove to have professional/expertise (game-related skills), they will have a more enjoyable experience (Pietruszka, 2016). This may not be the case in this study since some participants struggled by not knowing how to play the game. However, given that the game in question is the competitive type of game usually shown in eSports, perhaps individuals who watch these types of games may have different motivations (Sjöblom and Hamari, 2017). The results of our study indicate that, while individuals may be interested in watching gameplay footage, actually playing the game seems to be key for reducing stigma.

The results of this study show important implications for anti-stigma campaigns. Notably, it seems that having individuals being able to interact with a narrative space while taking on the role of a character suffering from a severe mental illness is able to ultimately reduce the desire to distance themselves from those with mental illnesses more generally. Thus, video games like the one utilized in this study may function as an anti-stigma intervention. While these results are preliminary in that it is unclear whether they would function the same way with different populations, they show early promise for the development of future campaigns.

The use of video game interventions like *Hellblade* may be useful in delivering anti-stigma messages primarily because they are not specifically designed to do so. In other words, the game was developed for commercial and aesthetic applications, not for the explicit purpose of reducing stigma. Thus, the game, and other games like it, may be less likely to activate reactance in players (see Moyer-Gusé, 2008); as additional studies replicate the findings shown here, the viability of various types of games as stigma-reduction interventions will become more apparent. Future study should be devoted to the power of commercial entertainment to bypass reactance in audience members.

## Limitations and Directions for Future Research

Of course, there are limitations to the current study that must be discussed. First, the selection of the game itself may have proved to be a challenge for participants who do not often play video games. The game does not have a dedicated tutorial and does not show participants how to use controls or where to go. Thus, there may have been some confusion for participants, making it somewhat difficult for them to play the game. Future research should replicate the results found here with other games with varying degrees of difficulty, particularly if the creation of interventions is the goal.

Further, a student sample was utilized for this study. Thus, the results cannot necessarily be generalized to a more general population. Future studies should bring in participants from various demographics to ensure that the results hold for other populations.

Another potential limitation for this study is that we only examined the short-term impacts of the video game as a potential intervention. As a result, we have no way of knowing whether the effects found here are persistent. Future studies should further explore with long-term follow-ups to determine the persistent effectiveness of video games as stigma-reduction interventions.

Finally, participants in this study only played the first 45 min of the game; this was done to ensure that even beginning players would be able to pick up the story and the game’s controls. However, this represents only the beginning of Senua’s journey, which sees her come to terms with her own mental illness toward the end of the game. Effect sizes may have been larger had participants undergone the entire journey with Senua. Thus, future studies should vary the character’s progression, possibly exploring both self-acceptance and self-stigma through the character, as these different types of journeys may have an impact on stigma reduction.

## Data Availability Statement

The raw data supporting the conclusions of this article will be made available by the authors, without undue reservation.

## Ethics Statement

The studies involving human participants were reviewed and approved by the Florida State University Institutional Review Board. The patients/participants provided their written informed consent to participate in this study.

## Author Contributions

AF, JS, and NS designed the study. AF, JS, NS, and NE collected data. AF conducted the structural equation model. AF, JS, NS, and NE wrote and approved the final manuscript. All authors contributed to the article and approved the submitted version.

### Conflict of Interest

The authors declare that the research was conducted in the absence of any commercial or financial relationships that could be construed as a potential conflict of interest.
